# Rethinking the Viability and Utility of Inhaled Insulin in Clinical Practice

**DOI:** 10.1155/2018/4568903

**Published:** 2018-03-07

**Authors:** Lutz Heinemann, Christopher G. Parkin

**Affiliations:** ^1^Science & Co, Kehler Str. 24, 40468 Düsseldorf, Germany; ^2^CGParkin Communications Inc., 932 Vista Lago Way, Boulder City, USA

## Abstract

Despite considerable advances in pharmacotherapy and self-monitoring technologies in the last decades, a large percentage of adults with diabetes remain unsuccessful in achieving optimal glucose due to suboptimal medication adherence. Contributors to suboptimal adherence to insulin treatment include pain, inconvenience, and regimen complexity; however, a key driver is hypoglycemia. Improvements in the PK/PD characteristics of today's SC insulins provide more physiologic coverage of basal and prandial insulin requirements than regular human insulin; however, they do not achieve the rapid on/rapid off characteristics of endogenously secreted insulin seen in healthy, nondiabetic individuals. Pulmonary administration of prandial insulin represents an attractive option that overcomes limitations of SC insulin by providing more a rapid onset of action and a faster return of action to baseline levels than SC administration of rapid-acting insulin analogs. This article reviews the unique PK/PD properties of a novel inhaled formulation that support its use in patient populations with T1D or T2D.

## 1. Introduction

Use of basal-bolus intensified insulin therapy (IIT) is considered standard of care for treatment of patients with type 1 diabetes (T1D) [1]. In type 2 diabetes (T2D), treatment with subcutaneous (SC) injections of long-acting basal insulin analogs is recommended as the first step when initiating insulin therapy even though postprandial hyperglycemia occurs early in disease progression due to the loss of early-phase insulin secretion [2]. Numerous studies have demonstrated significant clinical benefits of early initiation of IIT in individuals with T2D [3–10]. In addition to its immediate impact on lowering blood glucose (BG) levels, early insulin treatment has been shown to preserve beta cells and improve beta cell function [7, 9, 11, 12], and it may help protect against endothelial dysfunction and vascular disease [13, 14].

Despite considerable advances in pharmacotherapy, insulin delivery systems, and self-monitoring technologies in the last decades, a large percentage of adults with diabetes remain unsuccessful in achieving optimal glucose control, reflected by not reaching their HbA1c goals [15, 16]. Insufficient glucose control is associated with a higher risk of developing long-term diabetic complications, more frequent hospitalizations, higher healthcare costs, and elevated mortality rates [17–20]. A key contributor to poor glycemic control is suboptimal medication adherence, which is linked to a number of factors including psychosocial status, age, physical limitations, patient skills/knowledge, medication side effects, pain, inconvenience, complexity of insulin regimens, and cost [21, 22]. Needle phobia in a subgroup of patients with diabetes, which often goes unrecognized or unreported, contributes to patient inertia and manifests as resistance to starting insulin injections even as T2D progresses from insulin resistance to total insulin deficiency [23, 24]. Additionally, clinicians often do not initiate or intensify therapy appropriately for patients with diabetes, a condition often referred to as clinical inertia [25–32].

In many individuals with insulin-treated diabetes, their attempts to achieve their glycemic targets are associated with frequent and/or severe hypoglycemia (SH) [33–38]. The combination of excessive SH and hypoglycemia unawareness makes fear of hypoglycemia a key driver of suboptimal adherence [33, 39–41]. As a result, many of these individuals are reluctant or unable to follow and/or adjust their insulin regimens as needed. There is still great reluctance to start insulin therapy despite significant improvements in the pharmacokinetic (PK) and pharmacodynamic (PD) properties of SC insulin formulations and the development of easy-to-use insulin pens with finer needles that cause less pain. Many patients with T2D regard the needle as the last resort.

The insulin formulations available today enable a more physiologic approach for coverage of basal and prandial insulin requirements; however, the rapid on/rapid off characteristics of endogenously secreted insulin seen in healthy, nondiabetic individuals are still not possible to mimic. All insulin preparations for injection have to overcome the limitations of administration into the SC tissue. For example, the absorption rates of SC rapid-acting insulin analogs are not fast enough to match physiological needs [42]. The extended duration of insulin action seen with prandial insulins increases the risk of overcorrection (insulin stacking) when there is still significant insulin on board [43]. Many patients are frustrated by the high intraindividual variations in insulin action they experience, which are mainly driven by variations in blood flow and/or tissue hypertrophy at the injection/infusion site [42]. The limitations of SC insulin therapy for prandial glucose control put individuals with insulin-treated diabetes at risk for both postprandial hyperglycemia and late postprandial hypoglycemia [42, 44, 45].

In this review article, we discuss the unique PK/PD properties of a novel inhaled formulation, Technosphere® insulin (TI), approved by the U.S. Food and Drug Administration as Afrezza® (insulin human) inhalation powder and inhaler (MannKind Corporation, Westlake Village, CA), that support its use in patient populations with T1D or T2D. Pulmonary administration of TI as a prandial insulin is an attractive option that helps to overcome many of the limitations of SC insulin formulations. With its more rapid onset of action and faster return of action to baseline levels [46, 47], combined with lower intrapatient variability of insulin action [48, 49], TI therapy provides a unique PK/PD profile that approximates the on/off characteristics of normal prandial insulin secretion better than any SC therapy.

## 2. Evolution of Inhaled Insulin

Delivery of medications through the pulmonary system is used extensively in the treatment of respiratory diseases due to the unique characteristics of the lung. The distal lung presents a large, highly perfused surface area, which allows for rapid absorption of small particles (with a diameter between 1 and 3 *μ*m) into the systemic circulation [50]. An additional advantage of pulmonary drug delivery is that “first pass” metabolism (degradation of the drug in the liver) is avoided [51].

Sanofi-Aventis developed the first commercial inhaled insulin product (Exubera), which was approved by the FDA and European Medicines Agency (EMA) in 2006 and marketed by Pfizer [52]. Although Exubera offered the advantage of painless insulin administration by the pulmonary route of administration, its PK/PD characteristics were similar to SC injected rapid-acting insulin analogs and, thus, offered no additional clinical benefit in postprandial glycemic control [53]. Further, the inhaler device was large and the handling procedure for insulin administration was cumbersome [44, 54]. Pfizer withdrew Exubera from the market after two years when it failed to gain acceptance from patients and providers (e.g., low sales) [52, 55].

In 2014, Mannkind Corporation received U.S. regulatory approval for Afrezza, an inhaled insulin with ultrarapid PK/PD properties that enable improved postprandial glycemic control in adults with T1D or T2D. This inhaled insulin has a PK/PD profile with a rapid onset of action and a short duration of action that differentiates it from SC administered rapid-acting insulin analogs and earlier inhaled insulins [56].

## 3. Afrezza Inhalation System

The Afrezza Inhalation System is a drug/delivery device combination product consisting of Technosphere Insulin (human insulin) inhalation powder (TI) prefilled into single-use cartridges and the Afrezza inhaler.

### 3.1. Technosphere Insulin

TI is a dry powder formulation composed of recombinant human insulin adsorbed onto Technosphere microparticles formed by fumaryl diketopiperazine (FDKP) under mildly acidic conditions. FDKP undergoes intramolecular self-assembly and crystallizes into microparticles with a median diameter of approximately 2.0–2.5 *μ*m [57, 58]. This size range provides a near-optimal aerodynamic diameter for delivery to the deep lung. Larger particles tend to be deposited in the mouth, throat, or upper airways, while smaller particles may be exhaled rather than deposited in the alveoli [50, 51]. Because FDKP is highly soluble in water at neutral or basic pH, TI dissolves rapidly in the alveolar fluid of the deep lung. Insulin and FDKP are then rapidly absorbed into systemic circulation due to the combination of large surface area for absorption and the thin alveolar barrier between air and blood.

The toxicology program for FDKP includes carcinogenicity in two species (up to 2 years in rats), chronic toxicology in two species, reproductive toxicology, safety pharmacology, and genetic toxicology. FDKP is biologically inactive and functions solely as the particle matrix to deliver the insulin efficiently to the alveoli. It is not metabolized and is eliminated via the renal route [59]. Approximately 20% of FDKP is deposited in the throat, subsequently swallowed after inhalation [60], and eliminated in the feces; FDKP has negligible oral bioavailability. *In vitro* studies have found no evidence that FDKP is cytotoxic to the human lung and there was no indication of any effect on airway epithelial tight junction integrity, cell viability, or cell permeability [57].

### 3.2. TI Cartridges

TI is packaged in single-use, color-coded cartridge dosages: 4 units (blue), 8 units (green), and 12 units (yellow). The cartridge labels are based on a conservative conversion factor used in a phase 3 study [61] that was intended to reduce the risk of hypoglycemia during the transition from SC insulin to TI. Because the nominal cartridge dose tends to overestimate TI's metabolic effect, many patients need to up-titrate to achieve glycemic control.

### 3.3. Inhaler

The Afrezza inhaler is a key part of the drug delivery system and is essential to achieve consistent, reproducible insulin delivery. The discreet, thumb-sized device consists of purpose-built, plastic, injection-molded components assembled with an ultrasonic weld. For TI administration, the patient opens the inhaler, inserts the appropriate cartridge, and inhales. The inhalation effort lifts, deagglomerates, and disperses TI into the lungs. For doses above 12 units, successive inhalations from multiple cartridges are necessary. The device is low maintenance (discarded and replaced every 15 days) and requires no cleaning.

## 4. Pharmacokinetic/Pharmacodynamic (PK/PD) Properties

The PK/PD properties of TI have been assessed in two phase 1 hyperinsulinemic-euglycemic glucose clamp studies. The first was an open-label, randomized, four-way crossover study in 32 healthy subjects to evaluate dose proportionality and linearity, relative bioavailability, and PD response of TI delivered at four dosages (4, 12, 24, and 32 units) compared with 15 units of SC regular human insulin [62]. The primary PK parameters were area under the curve (AUC) and maximum serum insulin concentration (*C*_max_), which were calculated from C-peptide corrected PK measurements. A power-law analysis demonstrated dose proportionality for AUC_0–180_ (slope = 1.00, 90% CI:0.939–1.061) and AUC_0–inf_ (0.949, 0.880–1.019). *C*_max_ was slightly more than dose proportional (1.067, 1.013–1.120). Relative bioavailability was determined by dose-normalized AUC comparisons of the various TI doses to SC insulin. The median bioavailability of TI was approximately 24% relative to SC analog insulin. The PD effect was assessed by the glucose infusion rate (GIR) required to maintain euglycemia (90 ± 10 mg/dL) for 4 hours postdosing in this glucose clamp study. The GIR for TI reached a maximum within 30–50 minutes after TI dosing, whereas maximal metabolic effect was achieved 170–234 min after receiving SC analog insulin.

In the second study, inhalation of 8 units of TI was compared with SC injection of 8 units of insulin lispro in 12 patients with T1D [63]. Absorption of inhaled TI was again rapid: *C*_max_ (51 *μ*U/mL) occurred at a median *t*_max_ of 8 min compared with a maximum concentration of 34 *μ*U/mL occurring approximately 50 min after dosing with SC insulin lispro. Return to predose levels occurred at 180–240 minutes with TI versus approximately 280 minutes for SC insulin lispro.

**Figure 1:**
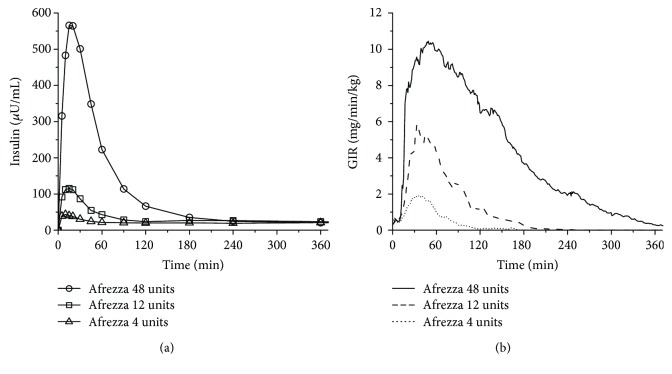
Pharmacokinetics (a) and pharmacodynamics (b) of TI after oral inhalation of 4, 12, or 48 units.

Current product labeling (prescribing information) reports the most recent view on the PK/PD properties of TI [46]. The updated label information is based on data from a randomized, controlled 6-way, crossover dose-response study comparing TI (4 units, 12 units, and 48 units) to SC insulin lispro (8 units, 30 units, and 90 units) in 30 patients with T1D [64]. The time to *C*_max_ again ranged from 10 to 20 min after inhalation of 4 to 48 units of TI; serum insulin concentrations declined to baseline by 60 to 240 min for these dose levels (Figure 1(a)). The onset of action with TI began after 12 min, reached the peak effect at 35–55 min, and declined to baseline by 90–270 min in a dose-dependent manner (Figure 1(b)). The time to GIR_max_ and duration of insulin effect were in contrast to SC insulin lispro: Median time to GIR_max_ was 90–180 min, and the effect lasted 360–660 min, also in a dose-dependent manner. Intrapatient variability in insulin exposure—measured by AUC and *C*_max_—was approximately 16% (95% CI 12–23%) and 21% (16–30%), respectively. Intrapatient variability in AUC GIR and GIR_max_ was approximately 28% (21–42%) and 27% (20–40%), respectively.

**Figure 2:**
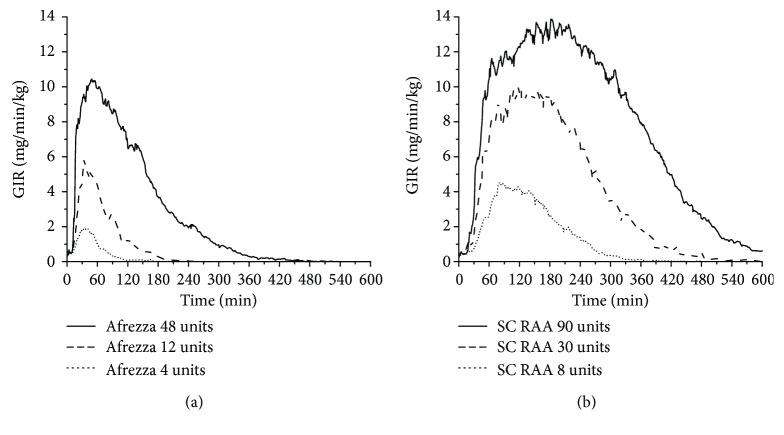
Peak glucose lowering effect of TI (Afrezza) after inhalation of 12 units (a) is similar to 8 units SC applied insulin lispro (RAA) (b) but with faster onset and shorter duration of action.

**Figure 3:**
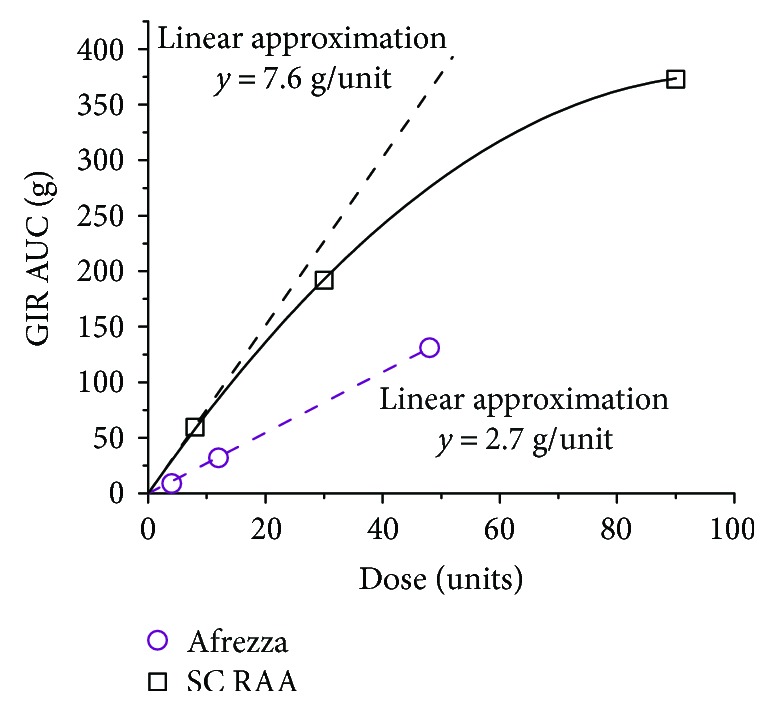
Total glucose effect: GIR AUC as a function of dose for TI (Afrezza) and SC lispro.

A comparison of glucose responses, as measured by GIR, showed a delayed onset with insulin lispro but similar peak effect between 12 units of TI (Figure 2(a)) and 8 units SC insulin lispro (Figure 2(b)). The total glucose effect per unit for TI is lower than for lispro due to its much more rapid absorption and return to baseline (Figure 3). In the linear portion of the GIR AUC curves, the conversion factor is approximately the ratio of slopes. These findings suggest that an appropriate approach to convert from prandial treatment with SC insulin may be to use 2.8 (=7.6/2.7) as a multiplier to calculate the initial Afrezza dosage. This is close to the ratio of 2.5 estimated by Baughman et al. based on the full dose–response curve [64]. Using the value from Baughman et al., 8 units SC rapid-acting insulin would be replaced by 20 units Afrezza. However, it is important to note that the glucose effect described in the PD evaluations is based on clamp studies, which may or may not equate to the dose equivalence in real situations. Therefore, using a smaller factor, such as 1.5 (replace 8 units SC insulin with 12 units Afrezza), may be advisable. However, rapid up-titration of dosages will likely be required to achieve optimal postprandial glycemic control [61, 65].

In a separate study using bronchoscopy with bronchoalveolar lavage, the concentration of insulin in lung declined rapidly following TI inhalation and was below limits of quantification (2 *μ*U/mL) by 12 hours [60]. FDKP concentration in the lung declined on the same time scale. The estimated clearance half-life from the lung of both insulin and FDKP was approximately one hour, suggesting the potential for TI accumulation on chronic administration is minimal.

## 5. Potential Safety Issues in Special Populations

### 5.1. Acute Bronchospasm, Asthma, and Chronic Obstructive Pulmonary Disease (COPD)

TI is contraindicated in patients with chronic lung disease such as asthma or COPD. Acute bronchospasm has been observed in patients with asthma and patients with COPD following administration of TI. In a study of subjects with asthma, this response was prevented by pretreatment with bronchodilators, but when asthmatic subjects were instructed to withhold their asthma medications and bronchodilator prior to taking TI, they experienced modest declines in mean FEV_1_ (approximately 400 mL) 15 minutes after dosing [46]. This modest decline recovered spontaneously in most subjects by 120 minutes. However, 5 of 17 (29%) subjects with asthma developed bronchoconstriction, wheezing, or asthma exacerbation after taking TI. These conditions were relieved with bronchodilator therapy.

### 5.2. Decreased Pulmonary Function

**Figure 4:**
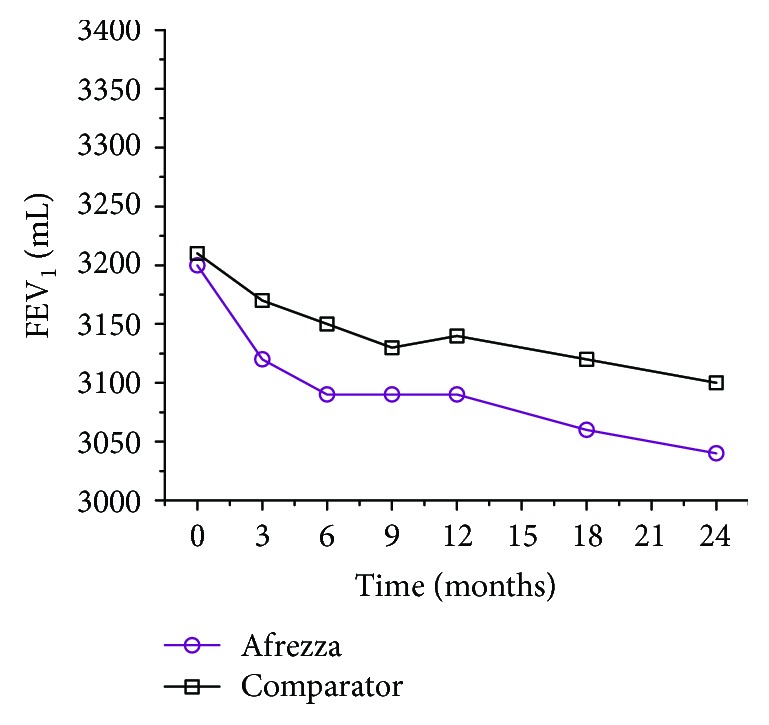
FEV_1_ as a function of time in the study.

Pulmonary function was closely monitored during development of TI. Analysis of the pooled pulmonary function test population (approximately 1500 patients in the TI and comparator groups) showed small declines from baseline in mean FEV_1_ at each time point [66]. The initial decline within the first 3 months was slightly steeper for the TI group but the curves remained parallel after that (Figure 4). A decline in FEV_1_ of ≥15% occurred in 6% of Afrezza-treated subjects compared to 3% of comparator-treated subjects. The least-squares mean difference between the groups was 36–45 mL from 3 months to 24 months, suggesting that the effect of TI on FEV_1_ is small and nonprogressive over 24 months of continued treatment. Some patients (315 from the TI groups and 334 from the comparator groups) participated in a safety extension study that demonstrated the difference in FEV_1_ disappeared within 1 month of resuming usual care [66]. Thus, changes in lung function associated with pulmonary insulin were small and nonprogressive and resolved after discontinuation.

### 5.3. Lung Cancer

Lung malignancies are of particular interest due to pulmonary administration. In clinical trials, no cases of lung cancer were seen in T1D subjects; however, 2 cases of lung cancer were reported in T2D subjects with a long history of cigarette exposure (1 in controlled trials, 1 in uncontrolled trials) for a total of 2 cases in 2750 patient-years; duration of Afrezza exposure was 1.75 years in one subject and 4 months in the other [66]. No cases of lung cancer were reported in the comparator groups (0 cases in 2169 patient-years). Two additional cases of lung cancer (squamous cell) in nonsmoking subjects who received TI were reported after the trials had been completed; cancers in these subjects were diagnosed 2+ years after exposure to Afrezza [66]. With the exceptions that both subjects were diabetic and both tumors were diagnosed as non-small cell/likely squamous, there was little else that these subjects had in common to show a pattern of increased risk. These data are insufficient to determine if TI has an effect on lung or respiratory tract tumors, but a causal association between use of TI inhalation powder and occurrence of new incident lung cancer is unlikely. TI is not contraindicated for patients with cancer or a prior history of cancer, but the physician and patient should consider whether the benefits outweigh this potential risk.

### 5.4. Upper Respiratory Tract Infection (URI)

Patients in one TI trial (*n* = 20) volunteered for a study to evaluate the effect of upper respiratory infections (URI) (e.g., common cold) on PK [67]. Subjects were tested during the infection and again after the infection had resolved. Neither exposure (AUC) nor absorption rate (*t*_max_) was significantly altered by URI. As with all insulin therapies, dosing adjustments might be required during an acute illness. The effect of TI on cough during a URI was not analyzed.

## 6. Findings from Clinical Studies

Two large clinical trials have studied the usage of TI in the treatment of patients with T1D and T2D [61, 65]. The first study was a 24-week, randomized, multicenter, noninferiority trial in patients with T1D that investigated the effects of TI in combination with basal insulin (SC long-acting insulin analogs) (*n* = 174) compared with patients treated with SC insulin aspart in combination with basal insulin (*n* = 171) [61]. Investigators also examined FEV_1_ in both groups as a measure of pulmonary safety. Both study groups showed comparable reductions in HbA1c, indicating similar improvements in glycemic control, which was the primary study outcome. A small between group difference (0.19 ± 0.086%, 95% CI 0.02–0.36) favored SC insulin therapy, but TI met the predefined clinical criterion for noninferiority. The frequency of hypoglycemic events was lower among patients on TI insulin than on SC insulin, especially in the period > 2–5 hours after meals. The hypoglycemic event rate was significantly lower among TI versus SC insulin users (9.8 versus 14.0 events/patient-month, respectively, *p* < 0.0001). In addition, patients on TI insulin experienced a small weight loss compared with a gain for patients on SC insulin (*p* = 0.0102). The most frequent adverse event among TI patients was cough (31.6%), which was mostly mild but led to discontinuation of 2.8% of patients. Importantly, the between-group difference for mean change from baseline in lung function parameters was small and disappeared upon TI discontinuation. For example, from a baseline value of 3.43 L, FEV_1_ declined by 0.07 L in the TI group and 0.04 L in the SC group. The between-group difference was approximately 30 mL.

The second study investigated the efficacy and safety of TI in insulin-naïve patients with T2D inadequately controlled on oral antidiabetes agents (OADs) [65]. This 24-week, randomized, double-blind, placebo-controlled trial randomized patients to TI treatment in combination with OADs (*n* = 177) or inhaled placebo with OADs. At 24 weeks, the TI group showed a significant reduction in HbA1c from baseline compared to the placebo group (−0.8% versus −0.4%, *p* < 0.0001), and more TI patients (38%) versus placebo patients (19%) achieved an HbA1c ≤ 7.0% (*p* = 0.0005). Postprandial hyperglycemia was more effectively controlled with TI treatment. As to be expected, the incidence of all hypoglycemic events in the TI group was higher than in patients with placebo (67.8% versus 30.7%, *p* < 0.0001); however, the difference in incidence of SH events was not significant (5.7% versus 1.7%, *p* = 0.0943). The most common adverse event was mild, transient dry cough. This occurred with similar frequency in both study groups (23.7% for TI; 19.9% for placebo) with the highest percentage of new-onset cough occurring within the first week of treatment (TI and placebo) and then diminishing over time. A small decline in FEV_1_ from the baseline value of 2.83 L was noted in both groups, with a slightly larger decline in the group receiving TI versus placebo (−0.13 for TI; −0.04 L for placebo). The difference was resolved after discontinuation of treatment.

## 7. TI Therapy in Clinical Practice

TI is indicated for use in adults (>18 years) with T1D and in adult patients with T2D who would benefit from the addition of prandial insulin to their current regimen, with or without basal insulin. TI should be administered at the beginning of a meal. No waiting time is required between doses if multiple cartridges are needed. Because upward titration of TI is often required, frequent BG monitoring or use of continuous glucose monitoring (CGM) is recommended. Patients should titrate their prandial insulin doses based on patterns revealed by BG testing after a meal.

### 7.1. Before Initiating TI Therapy

Given the risk of acute bronchospasm in patients with chronic lung disease, clinicians are advised to obtain a detailed medical history, perform a thorough physical examination, and conduct measurement of FEV_1_ prior to initiating TI therapy. TI is contraindicated in patients with asthma or COPD, and it should not be recommended to patients who smoke. FEV_1_ should be assessed again after 6 months of therapy and annually, even in the absence of pulmonary symptoms [46].

### 7.2. TI Initiation in Insulin Naive Patients

It is recommended that all individuals naïve to insulin therapy be started on 4 units of TI as a starting point. However, this is an extremely low starting dose and rapid upward titration to the appropriate prandial insulin dose will likely be required to maintain and improve postprandial glucose control. Safe titration will require regular postprandial glucose monitoring (see above).

### 7.3. Conversion from Subcutaneous Rapid-Acting Insulin Analogs to TI

PD studies have shown that an appropriate conversion ratio is approximately 2.5 units TI to 1.0 units SC rapid-acting insulin analog [64] (e.g., 20 units Afrezza = 8 units rapid-acting insulin analog). Patients should be advised to round up to the closest cartridge or cartridge combination. For example, conversion from 9 units SC of rapid-acting insulin would be performed as follows: multiply 9 units by 2.5, which equals 22.5 units TI, and round up to a final dose of 24 units (e.g. three 8-unit cartridges or two 12-unit cartridges). However, as a safety measure, it is advisable to use a lower conversion factor (e.g., 1.5 to 1.0) to obtain *starting* doses but rapid up-titration of dosages will likely be required. A ratio of 1.5 to 1.0 is used in these examples. No matter how the *initial* dose is selected, careful monitoring and dose adjustment/titration of prandial and basal insulin are required to reach satisfactory glucose control.

### 7.4. Determining Starting Dose for Patients Currently Using Subcutaneous Premixed Insulin

For individuals currently using premixed insulin preparations, the following formula can be used:

1. Divide one half of the total daily injected premixed insulin equally among three meals.
2. Convert each estimated injected mealtime dose to the appropriate TI dose (1.5 to 1.0 ratio).
3. Administer the other half of the total daily premixed dose as an injected basal insulin dose.

For example, if the current total daily dose of premixed insulin is 90 units, the mealtime TI dosage calculation would be based on 45 units of the injected insulin. The prandial dosage would be divided equally among the three daily meals (45 units ÷ 3), which would equate to 15 units of SC injected insulin per meal. Based on the dose conversion ratio, each mealtime dose would be 22.5 units TI, which would then be rounded up to 24 units to accommodate the cartridge options (six 4 unit cartridges, three 8 unit cartridges, two 12 unit cartridges, or any other combination that sums to 24 units). The basal insulin requirement would be covered by SC injection of 45 units of a long-acting insulin analog.

Patients on premixed insulins other than 50/50 can follow the directions above to obtain their starting doses with the understanding that the basal insulin may have to be adjusted if fasting glucose measurements are not in the target range. Basal/prandial starting ratios other than 50/50 could be used, but they were not tested in clinical trials.

### 7.5. Determining Starting Dose for Patients Currently Using Prandial Insulin with/without Basal Insulin

Because these patients are already using insulin therapy, it is appropriate to convert their current prandial dose to TI, using the 1.5 conversion ratio. Again, rapid up-titration will likely be necessary to achieve optimal postprandial control. It is important to monitor both postprandial glucose control and fasting BG, as it is often necessary to adjust basal insulin dosages once postprandial glucose levels are within target range.

## 8. Summary

Improvements in the PK/PD characteristics of today's SC insulins provide more physiologic coverage of basal and prandial insulin requirements than regular human insulin; however, they do not achieve the rapid on/rapid off characteristics of endogenously secreted insulin seen in healthy, nondiabetic individuals. As a result, patients often have difficulty achieving desired postprandial glucose control, and the long duration of SC insulin activity puts patients at increased risk of delayed postprandial hypoglycemia [42, 44, 45].

Although the most recent SC insulin formulation Fiasp® insulin aspart injection (Novo Nordisk, Plainsboro, NJ) may provide an onset of action nearly as rapid as TI, its duration of action is much longer [68]; however, this has not been evaluated in comparative studies. The risk for delayed postprandial hypoglycemia is not reduced relative to other rapid-acting insulin products, nor does Fiasp address adherence issues associated with subcutaneous insulin administration.

Pulmonary administration of TI addresses many of the limitations of SC insulin and has been shown to improve glycemia in insulin-naïve T2D individuals [65], consistently demonstrating less hypoglycemia than SC insulin [69]. In T1D, TI produced comparable HbA1c reductions with less hypoglycemia, especially 2–5 hours after the meal [61]. In summary, treatment with TI offers a safe and efficacious option for managing diabetes in patients with T1D and T2D.
